# OpenPhi: an interface to access Philips iSyntax whole slide images for computational pathology

**DOI:** 10.1093/bioinformatics/btab578

**Published:** 2021-08-06

**Authors:** Nita Mulliqi, Kimmo Kartasalo, Henrik Olsson, Xiaoyi Ji, Lars Egevad, Martin Eklund, Pekka Ruusuvuori

**Affiliations:** Department of Medical Epidemiology and Biostatistics, Karolinska Institutet, 171 77 Stockholm, Sweden; Department of Medical Epidemiology and Biostatistics, Karolinska Institutet, 171 77 Stockholm, Sweden; Faculty of Medicine and Health Technology, Tampere University, 33014 Tampere, Finland; Department of Medical Epidemiology and Biostatistics, Karolinska Institutet, 171 77 Stockholm, Sweden; Department of Medical Epidemiology and Biostatistics, Karolinska Institutet, 171 77 Stockholm, Sweden; Department of Oncology and Pathology, Karolinska Institutet, 171 64 Stockholm, Sweden; Department of Medical Epidemiology and Biostatistics, Karolinska Institutet, 171 77 Stockholm, Sweden; Faculty of Medicine and Health Technology, Tampere University, 33014 Tampere, Finland; Institute of Biomedicine, University of Turku, 20014 Turku, Finland

## Abstract

**Summary:**

Digital pathology enables applying computational methods, such as deep learning, in pathology for improved diagnostics and prognostics, but lack of interoperability between whole slide image formats of different scanner vendors is a challenge for algorithm developers. We present OpenPhi—Open PatHology Interface, an Application Programming Interface for seamless access to the iSyntax format used by the Philips Ultra Fast Scanner, the first digital pathology scanner approved by the United States Food and Drug Administration. OpenPhi is extensible and easily interfaced with existing vendor-neutral applications.

**Availability and implementation:**

OpenPhi is implemented in Python and is available as open-source under the MIT license at: https://gitlab.com/BioimageInformaticsGroup/openphi. The Philips Software Development Kit is required and available at: https://www.openpathology.philips.com. OpenPhi version 1.1.1 is additionally provided as Supplementary Data.

**Supplementary information:**

Supplementary data are available at *Bioinformatics* online.

## 1 Introduction

Pathology is transitioning into a digital discipline (Pantanowitz *et al.*, 2018). Digitization of microscopy slides into Whole Slide Images (WSI) has been feasible for over 20 years, but the milestone event of regulatory approval by the United States Food and Drug Administration (FDA) for the Philips Ultra Fast Scanner (UFS), based on non-inferiority to conventional microscopy (Mukhopadhyay *et al.*, 2018), is expected to promote the clinical adoption of the technology. Application of image analysis and deep learning on WSI data has enabled computational pathology, which aims at e.g. improved diagnosis and prognosis of diseases and reduction of inter-observer variability among pathologists (Bera *et al.*, 2019).

However, the nature of WSI data imposes challenges to developers. The multi-gigapixel WSIs frequently exceed memory capacity, a problem typically tackled by resolution pyramid approaches implemented in image formats specific to each scanner vendor. As a consequence, standard image processing libraries cannot be used with WSIs. The Digital Imaging and Communications in Medicine (DICOM) Standards Committee Working Group 26 has released a standard to improve the interoperability of WSIs (Singh *et al.*, 2011) which, despite its potential, has still not been widely adopted (Herrmann *et al.*, 2018). Reverse engineering of proprietary formats has resulted in vendor-neutral WSI libraries such as OpenSlide (Goode *et al.*, 2013). However, none of these libraries support the Philips iSyntax format (Hulsken, 2020). With increasing clinical use of the FDA approved Philips UFS, it is crucial for developers to have efficient ways of accessing the growing number of iSyntax WSIs. One approach for circumventing this issue is converting iSyntax to an open format (https://github.com/glencoesoftware/isyntax2raw), e.g. OME-TIFF (Besson *et al.*, 2019). However, conversion is problematic in the case of large datasets, since typically one is also required to archive the original data. Moreover, in view of clinical use, utilizing the native format of the scanner may be desirable due to regulatory considerations.

Philips recently released a Software Development Kit (SDK) for iSyntax, but several challenges remain. Firstly, the SDK represents a relatively low-level interface requiring familiarity with the non-standard iSyntax format. This not only slows down algorithm development, but also increases the risk of software bugs. Secondly, since iSyntax and the SDK are proprietary and closed source, they have thus far not been incorporated into libraries such as OpenSlide. This means developers cannot rely on a single vendor-neutral application programming interface (API) for compatibility across scanners, but need to customize software for Philips. To mitigate these problems, we present OpenPhi—Open PatHology Interface, an API that functions as a compatibility layer on top of the Philips SDK. OpenPhi works as a plug-in interface in Python, the language widely used by deep learning libraries and developers.

## 2 Methodology and key features

**Fig. 1.**
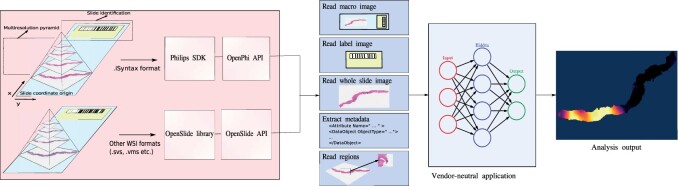
Left: WSIs contain: (1) a label image for sample identification, (2) a macro image of the glass slide and (3) a multiresolution pyramid of the high-resolution pixel data. OpenPhi provides a compatibility layer, facilitating access to iSyntax WSIs via the Philips SDK, while adhering to a unified API consistent with the OpenSlide Python API. Middle: streamlined access to the images and metadata is provided to developers by a set of high-level methods. Right: applications capable of analyzing WSIs in vendor-neutral manner can be built relying on the unified API.

The structure of OpenPhi follows the iSyntax data model (Fig. 1). The names, parameters and output of the API methods adhere to the OpenSlide Python API, allowing adapting existing vendor-neutral code for iSyntax compatibility by replacing OpenSlide with OpenPhi. OpenPhi itself has no strict requirements for the operating system or Python version. To meet the current requirements of the Philips SDK 2.0, we developed OpenPhi on Ubuntu 18.04 and Python 3.6.9. We verified the practical usability of OpenPhi by integrating it with a previously developed deep learning framework for Gleason grading of prostate biopsies (Ström *et al.*, 2020).

### 2.1 Reading label and macro images

The label image associated with an iSyntax file typically contains some form of sample identification numbers in a format specific to each clinical laboratory, and can be e.g. processed using optical character recognition algorithms to extract this information. The macro image, representing a low-resolution view of the glass slide acquired before scanning, may be utilized e.g. for coarse segmentation tasks or for troubleshooting purposes.

### 2.2 Reading regions of interest

The *read_region* method extracts pixel data from a rectangular region in the WSI at a desired resolution. Typically, only a limited amount of pixels are required at any given time by downstream applications, necessitating efficient random access across different locations and resolution levels. Unlike most WSI formats, iSyntax achieves this using a recursive Discrete Wavelet Transform to avoid storing downsampled copies of the image. The extracted pixel arrays can be used e.g. as input to deep neural networks.

### 2.3 Reading a whole slide image

The *get_thumbnail* and* read_wsi* methods read the entire WSI at desired maximum dimensions or a specified resolution, respectively. This is typically performed to obtain a more detailed view of the entire scanned area than the macro image. Typical use cases include segmentation of tissue regions and multi-step analysis with progressively increased resolution.

### 2.4 Extracting metadata

DICOM compliant metadata attributes are extracted as DICOM tags. All other metadata information are provided adhering to the OpenSlide generic properties format. Metadata properties include e.g. dimensions and pixel sizes of each resolution level, imaging parameters and device identifiers. The DICOM metadata tags can for example be utilized by developers building downstream applications capable of generating DICOM compliant output WSIs.

## 3 Conclusion

We present OpenPhi, an API that provides developers streamlined access to the Philips iSyntax image format. Building on the API, developers can implement algorithms with minimal effort spent on dealing with the intricacies of the proprietary format and on adapting existing vendor-neutral code. This can facilitate the computational pathology community to utilize the growing resources of WSI data stored in iSyntax format.

## Supplementary Material

btab578_Supplementary_Data
